# Food Behaviours and Health Indicators in Manitoba Adolescents and Relation to the Healthy Eating Index

**DOI:** 10.3390/ijerph20032007

**Published:** 2023-01-21

**Authors:** Joyce Slater, Bhanu Pilli, Aynslie Hinds, Alan Katz, Marcelo L. Urquia, Julianne Sanguins, Chris Green, Jaime Cidro, Dan Chateau, Nathan Nickel

**Affiliations:** 1 Department of Food and Human Nutritional Sciences, University of Manitoba, Winnipeg, MB R3T 2N2, Canada; 2 Department of Psychology, University of Winnipeg, Winnipeg, MB R3B 2E9, Canada; 3 Department of Community Health Sciences, Max Rady College of Medicine, University of Manitoba, Winnipeg, MB R3E 0W3, Canada; 4 Department of Family Medicine, Max Rady College of Medicine, University of Manitoba, Winnipeg, MB R3E 0W2, Canada; 5 Manitoba Metis Federation, Winnipeg, MB R3B 0 J7, Canada; 6 Winnipeg Regional Health Authority, Winnipeg, MB R3B 1E2, Canada; 7 Department of Anthropology, University of Winnipeg, Winnipeg, MB R3B 2E9, Canada; 8 Research School of Population Health, Australian National University, Canberra, ACT 0200, Australia

**Keywords:** nutrition, eating behaviours, diet, adolescent, Manitoba, Canada

## Abstract

Adolescence is a vital period of growth and development, both of which are dependent on adequate nutrition; however, concerns persist about poor nutrition and inappropriate food behaviours. In addition to nutrition assessment, the context of food and health behaviour is necessary to understand how dietary choices are shaped and related to diet quality. This study describes food-related behaviours and health indicators associated with dietary quality among adolescents in Manitoba, Canada. A stratified two-stage sampling method was used to collect data on the diet, food behaviours and health indicators of 1587 grade nine students. Diet quality was analysed using the Healthy Eating Index-Canada. Several food behaviours and health indicators varied by gender and school region (urban, rural, northern). The Independent Samples t-test and one-way ANOVA (analysis of variance) assessed differences between groups on the Healthy Eating Index-Canada. Higher Healthy Eating Index-Canada scores were found for those eating family dinners more frequently; consuming breakfast and lunch more frequently; consuming breakfast at home; eating lunch and morning snacks at school; purchasing fewer meals and snacks from cafeterias and vending machines; believing that food and nutrition education is important; not attempting to lose weight; being classified as ‘healthy weight’; and getting more sleep. Many Manitoba youth are exhibiting food and health behaviours that increase their risk of having a poor diet.

## 1. Introduction

Adolescence is a critically important period of growth and development [1]; however, concerns about obesity and poor nutritional status in adolescence continue to grow among public health authorities [2,3]. In Canada, 12% of children aged 5–17 are obese and 19% are overweight [4]. Poor diet is associated with excess weight and the excessive consumption of highly processed, high fat and sugar foods [5,6,7]. These foods are ubiquitous in Canada, with the highest proportion being consumed by children and adolescents [8]. They are readily available, high in calories and tend to have low nutrient density [9]. Diets high in ultra-processed foods (e.g., convenience foods, sweet and salty snacks, fast food) are also associated with increased risk of obesity and all-cause mortality and numerous illnesses including cardiovascular diseases, cancers and respiratory diseases [10]. In adolescents, these diets are associated with cardiometabolic risks and asthma as well as poor mental health outcomes [11,12]. 

Dietary intake is contextualized in food behaviours. Many adolescents engage in food-related behaviours that contribute to their nutritional risk. These include meal skipping and weight loss dieting, which are associated with lower diet quality [13]. A low frequency of family meals is associated with a less healthy dietary intake, such as lower fruit and vegetable consumption and higher sugar-sweetened beverage intake [14]. Adolescents are also avid fast-food consumers compared to other age groups [15]. 

Consuming foods prepared outside the home, such as snacks and lunches purchased at school or take-away establishments, may present additional behavioural risk factors for adolescents who are increasingly making more independent food choices. Foods purchased away from home tend to have lower nutrient density and regular consumption is associated with lower diet quality and weight gain [15,16]. 

Adolescents’ food-related behaviours are also influenced by their families and peers. In addition to environments that promote highly processed and fast-food choices [8], parental eating habits are important influences on behaviours that persist into adulthood [17]. Youth also feel pressure to achieve and/or maintain a particular body size based on perceived standards so as to be accepted [18]. Consequently, they may engage in attempts to lose, or gain, weight to fit these ideals. This may involve food-related behaviours such as restrictive dieting or the use of supplements such as protein drinks [19]. This is mediated by gender, with females feeling pressure to be thin and fit, while males are encouraged to look fit and muscular [20,21].

Food-related behaviours can synergistically impact health through other health behaviours. Sleep is known to impact diet and weight, where insufficient sleep is associated with poor diet and obesity [22]. Poor diet and poor sleep each result in lower self-rated health; a combination of the two is associated with poorer self-rated health [23]. While many adolescents meet sleep recommendations, in Canada just over one third do not [24].

Canada is a sparsely populated and geographically widespread country; therefore, it is important to consider regional differences in food-related behaviours and health indicators. In Manitoba, different regions of the province have different socio-economic environments. For example, northern communities are remote and experience high rates of poverty and food insecurity due to high unemployment and very expensive food due to high transportation costs [25,26,27].

Food behaviours are important to understand within the context of diet, in order to plan appropriate strategies to improve nutritional health, but few studies have examined food behaviours and health indicators in Canadian youth and their relationship to diet quality. 

Understanding behavioural factors associated with diet quality is key to developing interventions that go beyond exhorting youth to “eat better”. The purpose of this study, therefore, was to describe food-related behaviours and health indicators associated with dietary quality among a population of grade nine students (13–16 years of age) in Manitoba, Canada. This study is part of a larger research study, ‘Food and Nutrition Security for Manitoba Youth (FANS)’ that collected information on dietary intake, food security, food-related behaviours, health indicators and body mass index; other publications are available and forthcoming [28].

## 2. Materials and Methods

The study was conducted according to the guidelines of the Declaration of Helsinki, and approved by the Joint Faculty Research Ethics Board at the University of Manitoba (protocol code HS21666 J2018:040). 

### 2.1. Participants

The Food and Nutrition Security for Manitoba Youth (FANS) study included a self-administered web-based survey that assessed dietary intakes, food behaviours and health-related indicators of grade nine students attending public school in the province of Manitoba, Canada. Eighteen of 37 school divisions were approached, with 14 agreeing. Within the agreeing divisions, classes with 10 or more grade nine students were invited to participate. Parents of participating students signed written consent forms and students provided assent at the start of the survey. A detailed description of the study design and rationale is reported elsewhere including demographic information on participants [28]. 

School divisions and schools were classified into northern, rural and urban regions of the province: school divisions within the Winnipeg Health Region are urban; divisions in the Northern Health Region are classified as northern; and remaining divisions are considered rural [29]. 

### 2.2. Measures

Dietary intake was assessed using a single 24 h dietary recall as part of the WEB-Q (Waterloo Eating Behaviour Questionnaire), a validated online tool for measuring the food and nutrient intake of adolescents [30]. All surveys were completed in the classroom during a weekday.

Diet quality was assessed using a Healthy Eating Index, a measure for assessing how closely an individual’s diet, or set of foods consumed, aligns with dietary guidelines [31], such as Canada’s Food Guide (2007) [32]. This study used a modified Canadian version of the Healthy Eating Index (HEI-C) [33]: ‘low fat vitamin-D fortified milk and dairy-free beverage alternatives’ and ‘seafood and plant-based proteins’ were criteria added to align with newer Canadian dietary recommendations [34]. Mean nutrient intakes and food group intakes were used to compute the HEI-C score (0–100), the main outcome variable.

Using the online WEB-Q, food behaviour was assessed via questions about frequency of meal consumption, meals consumed with family members and food purchasing habits. Health indicators included questions about eating-related weight control and sleep behaviours. Self-reported health and life satisfaction measures were included [35,36]. Body mass index (BMI) was calculated using self-reported height and weight, and classified using World Health Organization z-scores [37].

Students self-identifying as ‘other’ gender were not included in analyses because of small numbers, and because the HEI-C calculation uses sex-specific (male and female) dietary guidelines (32 and 33).

### 2.3. Statistical Analysis

Study data were analysed using SAS Version 9.4 (SAS Institute Inc., Cary, NC, USA) (variable derivation) and IBM SPSS Statistics for Windows Version 27.0 (IBM Corp., Armonk, USA) (tables and statistical outputs) statistical software packages. Chi-square test was used to determine significant associations and follow-up pairwise comparisons were carried out using Bonferroni correction for food behaviour and health indicator variables, by sex and region. Fisher–Freeman–Halton Exact test was performed with BMI classification categories by region due to low expected cell counts for underweight participants. For continuous descriptive data, a Shapiro–Wilk test was used to confirm normality. Independent samples t-test or one-way ANOVA with Tukey’s honestly significant difference (HSD) post hoc test was conducted with 2 and 3 comparison groups, respectively, to determine differences in mean HEI-C scores. Statistical significance was accepted at *p* ≤ 0.05. All analyses used unweighted sample data.

## 3. Results

### 3.1. Recruitment and Participants

The participating students represented 37 schools from 14 school divisions across Manitoba. Of the 1587 students who participated, 8%, 14% and 78% were from northern, rural and urban regions of the province, respectively. Half (50.5%) self-identified as female and 44.7% self-identified as male; 4.8% identified as “other” or did not disclose their sex.

### 3.2. Food Behaviours and Health Indicators

Just over three-quarters of students had dinner with their family 5–7 days per week (Table 1); however, almost one in ten dined with family only ‘once per week’ or ‘never’. Slightly more males than females dined with family most days. There was no difference in family dinner frequency by region.

Almost two-thirds of students ate breakfast 5–7 days per week while over 80% had lunch this frequently. More males than females ate breakfast and lunch regularly. Significantly fewer northern students ate breakfast regularly (half) compared to other regions. Lunch frequency did not vary by region.

The majority of students consumed breakfast and dinner at home and lunch at school. Slightly more males than females breakfasted at home. Daytime snacks were mainly consumed at school while most had evening snacks at home. Most (86%) rural students ate lunch at school while 44% of northern students had lunch at home.

Fewer than one in five students purchased food from a cafeteria more than twice a week while only 6% made purchases from vending machines this frequently. There were no differences between males and females with regard to cafeteria and vending purchases. Significantly more northern students (26%) than students in other regions ate cafeteria food more than twice a week while rural students made the most vending purchases.

Significantly more females (68%) than males (55%) had taken a food and nutrition course in school. Rural students had the highest participation in food and nutrition classes: 72% compared to 50% in northern students. Over 80% of students agreed that learning about food and nutrition in school is important.

Self-reported health varied by sex: 68% of males reported excellent/very good health compared to 55% of females.

Almost one third of females reported attempting to lose weight compared to 19% of males; however, almost one quarter of males were trying to gain weight compared to 9% of females. More urban students were trying to gain weight than in other regions.

The majority of students were classified as having a healthy weight, while significantly more males were classified as overweight and obese [28]. Weight classification varied by region, with significantly more students classified as overweight in northern and rural regions compared with urban.

**Figure 1:**
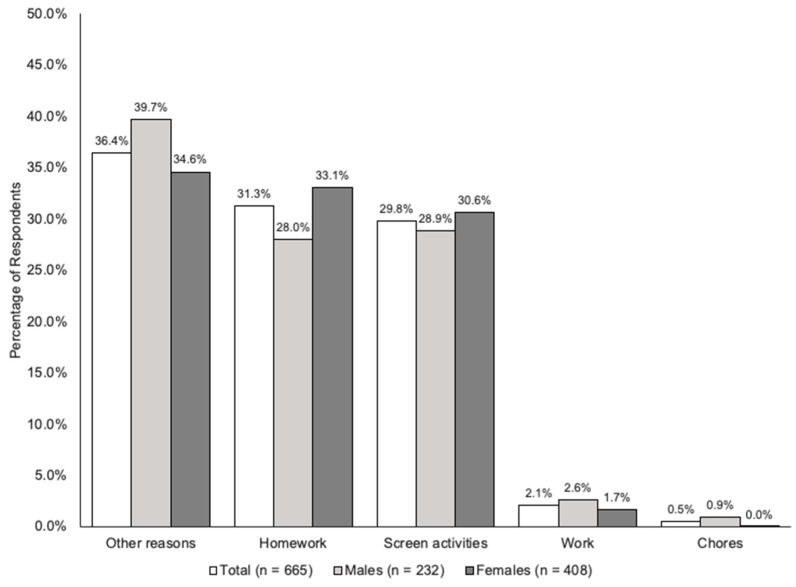
Main reasons for not getting sufficient hours of sleep.

Almost half (46%) of students reported that they did not get enough sleep regularly, with half getting seven hours or fewer on the previous night. Significantly more females than males did not get enough sleep, with eight percent of females getting less than six hours per night regularly. Significantly more northern students had less than six hours of sleep the previous night, and twice as many northern students (11%) regularly slept less than six hours per night compared with rural students, who slept the most. The main reasons for insufficient sleep were homework, screen activities and ‘other reasons’ (Figure 1).

### 3.3. Association with Healthy Eating Index-Canada

Several food behaviours and health indicators showed significant differences in mean Healthy Eating Index-Canada (HEI-C) scores (Table 2). Students who ate dinner with family members less than two days per week had significantly lower mean HEI-C scores than those sharing family dinners two or more days per week. Consuming breakfast and/or lunch 0–4 days per week also yielded significantly lower HEI-C scores. Students who consumed breakfast at home had higher HEI-C scores than those eating breakfast away from home, while those who ate lunch at school or other locations had higher scores than those eating lunch at home. Morning snacks at school were associated with higher HEI-C scores than those eaten at home; there were no differences in scores for the location of dinner or afternoon/evening snacks.

Purchasing meals and snacks from cafeterias and vending machines more frequently was associated with lower HEI-C scores. Students who felt learning about food and nutrition in school was important had higher HEI-C scores, as did students reporting excellent or very good health.

Students attempting to lose weight had significantly lower HEI-C scores than those not trying to lose weight; trying to gain weight did not yield different scores than those not trying to gain weight.

Students classified as overweight or obese had significantly lower HEI-C scores than those classified as healthy weight.

Students who got the most sleep per night (8–10 h) had significantly higher HEI-C scores than students getting 7 h or less. Those who usually slept less than six hours had the lowest HEI-C scores.

## 4. Discussion

This study explored the food behaviours and health indicators of grade nine adolescents, and their relationship to diet quality. It identified several food behaviours and health indicators associated with poor diet quality as measured by the Healthy Eating Index-Canada. 

While the majority of students reported having regular dinners with families, almost ten percent rarely had family dinners (i.e., never or once per week). Further, those having the fewest family dinners had significantly lower diet quality. This is consistent with other studies showing that family meals are associated with higher diet quality (e.g., eating more fruits and vegetables) and healthful meal patterns during adolescence [38,39]. Family meals have other positive benefits including associations with better academic achievement, well-being and fewer behavioural issues [40].

Regular breakfast and lunch consumption was observed in most males, but fewer females. This may be part of weight regulation for some female participants, as 30% reported attempting to lose weight. Breakfast was less frequently consumed in the northern region, where half of students only ate breakfast 0–4 days per week. This could in part be due to household food insecurity, which is significantly greater in northern and remote communities in Canada [26]. Those consuming breakfast regularly had higher HEI-C scores, indicative of better diet quality. This is consistent with the literature showing that breakfast eaters have greater micronutrient intake than breakfast skippers [41]. Breakfast skipping is associated with being overweight or obese [42], and adolescents who skip main meals have lower fruit and vegetable intake [43].

Regional differences in meal/snack purchases at school through vending machines and cafeterias may be due to the availability of these amenities. Northern students purchase food at school least frequently, possibly because food is inaccessible or unaffordable. Northern Manitoba has higher rates of unemployment and lower household income than other regions of the province [44].

Most students, regardless of gender and geography, felt that taking a school-based food and nutrition course was important; however, almost one half of males and one third of females had not taken a course. This may be because home economics classes, which traditionally teach this subject, are not available or mandatory in all Manitoba school divisions [45]. This reflects a trend observed across Canada and elsewhere, where there has been a decrease in home economics programs in recent decades [46]. 

One quarter of students were attempting to lose weight, although 50% more females were doing so than males. Conversely, almost three times the number of males were trying to gain weight. These patterns reflect normative gendered expectations of body shape, including thinness for women [18] and muscularity for men [47]. Adolescents have been shown to internalize social media messages reinforcing these ideals, which may have negative mental health effects in some [48]. It would also be important to explore the relationship between these beliefs and taking food and nutrition courses.

Body mass index varied regionally, with significantly more students residing in northern and rural communities classified as overweight compared with urban students. This may be in part due to reduced access to healthy foods, and higher food prices [49]. Healthier foods such as milk, fruit and vegetables have higher transportation costs, which are reflected in the grocery aisle [50,51,52]. These foods may be replaced by ‘other’ foods (e.g., high fat/salt/sugar convenience foods, sugar-sweetened beverages) which are more shelf-stable and less expensive than fresh alternatives, yet contribute to poor diet and weight gain. This was reflected in the lower HEI-C scores for those classified as overweight and obese.

Although adolescents have a reputation for excessive sleeping, almost half the participants felt they did not get enough sleep regularly with fewer females than males regularly getting more than eight hours of sleep per night, and almost one in ten getting less than six hours. These results are consistent with other studies that have found higher sleep deprivation in adolescent girls [53]. Screen activities and homework were the most frequently reported reasons for poor sleep in this study, which is consistent with other reports [54]. Poor sleep habits in adolescents are associated with poor health outcomes and health behaviours including poor dietary choices [55].

### Limitations

There are several limitations with this study. The sample was not representative of all adolescents in Manitoba due to the complex nature of provincial school board structures, and the voluntary nature of participation at the school division, school, classroom and student levels. It was also limited by those who chose to come to school on the day of data collection. Self-reported dietary intakes are subject to under-reporting through the omission of food intake [56] and portion sizes may be inaccurate due to reliance on memory [57]; however, the use of a multi-pass technique in the WEB-Q tool including photographic images for portion sizes and prompts for complementary foods helped address these limitations [30]. The BMI calculations used self-reported height and weight, which have been found to be misreported in surveys; therefore, those in the overweight and obese categories could be underestimated [58]. Further, the food behaviours and health indicators used are not exhaustive. Other variables could provide more in-depth context into youth nutrition and health including physical activity and screen time. Finally, the study did not include schools in First Nation communities, where food costs and unemployment are high and incomes are low compared to other communities in Manitoba [59,60]. Consequently, the results presented for the north region may over- or underestimate some variables. There was, however, participation by schools from all regions of the province, and the results are consistent with those observed in other Canadian regions [61]. Despite these limitations, the study did include a large sample from three regions of the province. Dietary intakes estimated through the validated tools are appropriate for the types of analyses conducted. Further studies should examine additional behaviours impacting diet quality, and qualitative studies could provide further insights into youth experiences of food, nutrition and eating, and contribute to appropriate intervention development.

## 5. Conclusions

This study indicates that food and health behaviours were associated with diet quality in the study population; however, these were mediated by gender and the region of residence. Similar patterns were observed for many behaviours across participant sub-groups (gender and region), and average HEI-C scores were poor, suggesting universal strategies to improve food behaviours and diet are required; however, the results also demonstrated that some targeted interventions may be necessary based on gender and region. In particular, strategies to address food insecurity and support school-based feeding programs in the north should be considered. Any intervention strategies need to be supported by multi-faceted policies and extend across sectors including education, food systems, health, social welfare and digital media [62]. 

## Figures and Tables

**Table 1:** Food behaviours and health indicators according to sex and region of participants.

Food Behavior or Health Indicator	Total	Sex			Region			
		Male *n* (%)	Female *n* (%)	*p-*Value	Norther n *n* (%)	Rural *n* (%)	Urban *n* (%)	*p-*Value
Family dinner frequency								
0–1 days/week	136 (9.2)	58 (8.8)	71 (9.4)	0.018 *	11 (8.7)	14 (6.7)	111 (9.7)	0.067
2–4 days/week	215 (14.5)	78 (11.8 ^a^)	128 (16.9 ^b^)		22 (17.3)	19 (9.1)	174 (15.1)	
5–7 days/week	1134 (76.4)	524 (79.4 ^a^)	557 (73.7 ^b^)		94 (74.0)	175 (84.1)	865 (75.2)	
Meal Frequency								
Breakfast								
0–4 days/week	591 (38.7)	194 (28.4 ^a^)	369 (47.7 ^b^)	<0.001 *	65 (49.6 ^a^)	83 (38.4 ^b^)	443 (37.5 ^b^)	0.026 *
5–7 days/week	938 (61.3)	490 (71.6 ^a^)	405 (52.3 ^b^)		66 (50.4 ^a^)	133 (61.6 ^b^)	739 (62.5 ^b^)	
Lunch								
0–4 days/week	262 (18.4)	89 (13.8 ^a^)	161 (22.7 ^b^)	<0.001 *	24 (19.8)	35 (17.9)	203 (18.4)	0.903
5–7 days/week	1160 (81.6)	556 (86.2 ^a^)	548 (77.3 ^b^)		97 (80.2)	161 (82.1)	902 (81.6)	
Previous day meal/snack location								
Breakfast								
Home	1308 (91.0)	614 (92.9 ^a^)	633 (89.0 ^b^)	0.013 *	105 (92.1)	187 (92.6)	1016 (90.6)	0.592
School/Other	130 (9.0)	47 (7.1 ^a^)	78 (11.0 ^b^)		9 (7.9)	15 (7.4)	106 (9.4)	
Morning snack								
Home	434 (35.5)	232 (42.2 ^a^)	181 (29.6 ^b^)	<0.001 *	37 (38.1 ^a^)	38 (24.2 ^b^)	359 (37.1 ^a^)	0.006 *
School/Other	787 (64.5)	318 (57.8 ^a^)	430 (70.4 ^b^)		60 (61.9 ^a^)	119 (75.8 ^b^)	608 (62.9 ^a^)	
Lunch								
Home	343 (22.9)	161 (23.8)	166 (22.1)	0.443	58 (44.3 ^a^)	30 (14.5 ^b^)	255 (22.0 ^c^)	<0.001 *
School/Other	1153 (77.1)	516 (76.2)	586 (77.9)		73 (55.7 ^a^)	177 (85.5 ^b^)	903 (78.0 ^c^)	
Afternoon snack								
Home	693 (54.4)	323 (55.9)	339 (53.3)	0.367	49 (51.6)	97 (54.5)	547 (54.7)	0.843
School/Other	580 (45.6)	255 (44.1)	297 (46.7)		46 (48.4)	81 (45.5)	453 (45.3)	
Dinner								
Home	1400 (91.9)	633 (92.8)	702 (91.1)	0.219	126 (96.9)	195 (90.7)	1079 (91.6)	0.083
School/Other	123 (8.1)	49 (7.2)	69 (8.9)		4 (3.1)	20 (9.3)	99 (8.4)	
Evening snack								
Home	1219 (93.0)	560 (94.1)	607 (92.4)	0.225	106 (93.8 ^a,b^)	158 (87.3 ^b^)	955 (93.9 ^a^)	0.005 *
School/Other	92 (7.0)	35 (5.9)	50 (7.6)		7 (6.2 ^a,b^)	23 (12.7 ^b^)	62 (6.1 ^a^)	
Frequency of school meal/snack purchases from cafeteria								
0–1 times/week	1261 (83.6)	563 (83.7)	642 (84.1)	0.802	96 (74.4 ^a^)	167 (79.1 ^a^)	998 (85.4 ^b^)	0.001 *
2–7 times/week	248 (16.4)	110 (16.3)	121 (15.9)		33 (25.6 ^a^)	44 (20.9 ^a^)	171 (14.6 ^b^)	
Frequency of school meal/snack purchases from vending machines								
0–1 times/week	1396 (94.3)	622 (94.0)	711 (95.1)	0.366	124 (97.6 ^a^)	190 (91.3 ^b^)	1082 (94.4 ^a,b^)	0.050 *
2–7 times/week	85 (5.7)	40 (6.0)	37 (4.9)		3 (2.4 ^a^)	18 (8.7 ^b^)	64 (5.6 ^a,b^)	
Self-reported health								
Excellent/Very good	910 (60.6)	452 (67.9 ^a^)	418 (54.6 ^b^)	<0.001 *	68 (53.1)	137 (64.0)	705 (60.8)	0.131
Fair/Poor	591 (39.4)	214 (32.1 ^a^)	348 (45.4 ^b^)		60 (46.9)	77 (36.0)	454 (39.2)	
Intentional weight loss								
Not really/not trying to lose weight	1130 (74.6)	546 (81.0 ^a^)	538 (69.7 ^b^)	<0.001 *	99 (78.0)	154 (71.6)	877 (74.8)	0.412
Yes/kind of trying to lose weight	385 (25.4)	128 (19.0 ^a^)	234 (30.3 ^b^)		28 (22.0)	61 (28.4)	296 (25.2)	
Intentional weight gain								
Not really/not trying to gain weight	1264 (84.3)	515 (76.9 ^a^)	696 (91.1 ^b^)	<0.001 *	110 (88.7 ^a,b^)	190 (90.5 ^b^)	964 (82.7 ^a^)	0.006 *
Yes/kind of trying to gain weight	236 (15.7)	155 (23.1 ^a^)	68 (8.9 ^b^)		14 (11.3 ^a,b^)	20 (9.5 ^b^)	202 (17.3 ^a^)	
Body Mass Index category								
Underweight	37 (3.0)	26 (4.2 ^a^)	11 (1.7 ^b^)	<0.001 *	3 (2.8 ^a^)	5 (2.7 ^a^)	29 (3.0 ^b^)	0.008 *^,E^
Healthy weight	895 (72.1)	410 (67.0 ^a^)	485 (77.0 ^b^)		62 (57.9 ^a^)	123 (67.6 ^a,b^)	710 (74.5 ^b^)	
Overweight	218 (17.6)	121 (19.8 ^a^)	97 (15.4 ^b^)		30 (28.0 ^a^)	37 (20.3 ^a,b^)	151 (15.8 ^b^)	
Obese	92 (7.4)	55 (9.0 ^a^)	37 (5.9 ^b^)		12 (11.2)	17 (9.3)	63 (6.6)	
Sleep: Getting enough sleep regularly								
Yes	795 (54.1)	424 (64.2 ^a^)	330 (44.4 ^b^)	<0.001 *	65 (53.3)	125 (59.8)	605 (53.1)	0.200
No	675 (45.9)	236 (35.8 ^a^)	413 (55.6 ^b^)		57 (46.7)	84 (40.2)	534 (46.9)	
Sleep: Hours previous night								
8–10 h	788 (51.3)	401 (58.7 ^a^)	350 (44.8 ^b^)	<0.001 *	53 (40.2 ^a^)	133 (61.6 ^b^)	602 (50.6 ^c^)	0.001 *
6–7 h	582 (37.9)	225 (32.9 ^a^)	333 (42.6 ^b^)		57 (43.2 ^a^)	63 (29.2 ^b^)	462 (38.9 ^a^)	
<6 h	167 (10.9)	57 (8.3 ^a^)	99 (12.7 ^b^)		22 (16.7 ^a^)	20 (9.3 ^b^)	125 (10.5 ^b^)	
Sleep: Usual duration per night								
8–10 h	827 (54.3)	421 (62.3 ^a^)	369 (47.4 ^b^)	<0.001 *	62 (48.8 ^a^)	143 (65.9 ^b^)	622 (52.8 ^a^)	0.001 *
6–7 h	594 (39.0)	221 (32.7 ^a^)	348 (44.7 ^b^)		51 (40.2 ^a^)	62 (28.6 ^b^)	481 (40.8 ^a^)	
<6 h	102 (6.7)	34 (5.0 ^a^)	62 (8.0 ^b^)		14 (11.0)	12 (5.5)	76 (6.4)	
Food and nutrition course in school								
Yes	912 (62.3)	358 (55.4 ^a^)	507 (67.6 ^b^)	<0.001 *	62 (50.4 ^a^)	151 (71.6 ^b^)	699 (61.9 ^c^)	<0.001 *
No	552 (37.7)	288 (44.6 ^a^)	243 (32.4 ^b^)		61 (49.6 ^a^)	60 (28.4 ^b^)	431 (38.1 ^c^)	
Importance of learning about food and nutrition in school								
Strongly agree/Agree	1183 (82.0)	526 (81.7)	608 (82.9)	0.537	100 (80.0)	160 (79.2)	923 (82.8)	0.393
Neutral/Disagree/Strongly disagree	259 (18.0)	118 (18.3)	125 (17.1)		25 (20.0)	42 (20.8)	192 (17.2)	

* *p* < 0.05: Chi-squared test for differences by sex and region. ^a,b,c^ Values in a row with unlike letters are significantly different. ^E^ Use with caution (due to small numbers in ‘underweight’ category).

**Table 2:** Comparisons of HEI-C scores by food behaviours and health indicators.

Food Behavior or Health Indicator	HEI-C Scores					
	n	Mean	SD	Median	Interquartile Range	p-Value
Family dinner frequency						
0–1 days/week	122	51.5 ^a^	10.9	51.2	14.4	<0.001 *
2–4 days/week	203	56.5 ^b^	11.0	56.3	15.0	
5–7 days/week	1058	57.0 ^b^	11.2	56.8	15.2	
Meal Frequency						
Breakfast						
0–4 days/week	547	53.7 ^a^	11.0	53.6	14.2	<0.001 *
5–7 days/week	875	58.0 ^b^	11.0	57.8	14.9	
Lunch						
0–4 days/week	241	53.5 ^a^	11.8	54.3	16.0	<0.001 *
5–7 days/week	1080	57.0 ^b^	11.1	57.0	15.2	
Previous day meal/snack location						
Breakfast						
Home	1219	57.1 ^a^	11.0	56.9	15.0	0.005 *
School/Other	121	54.1 ^b^	11.8	54.9	13.7	
Morning snack						
Home	397	55.9 ^a^	10.6	56.0	15.1	0.001 *
School/Other	736	58.2 ^b^	11.0	57.7	14.9	
Lunch						
Home	316	54.4 ^a^	11.6	55.1	16.2	<0.001 *
School/Other	1080	57.1 ^b^	10.9	56.6	14.7	
Afternoon snack						
Home	645	56.8	11.2	56.6	15.5	0.461
School/Other	541	57.3	10.8	56.7	14.0	
Dinner						
Home	1306	56.6	11.2	56.4	14.8	0.314
School/Other	114	55.4	11.3	54.4	17.4	
Evening snack						
Home	1141	57.0	11.0	56.8	14.8	0.347
School/Other	81	55.7	11.4	55.4	17.7	
Frequency of school meal/snack purchases from cafeteria						
0–1 times/week	1176	57.0 ^a^	11.2	56.9	15.4	<0.001 *
2–7 times/week	226	53.7 ^b^	10.7	53.6	14.1	
Frequency of school meal/snack purchases from vending machines						
0–1 times/week	1303	56.7 ^a^	11.2	56.5	15.2	0.005 *
2–7 times/week	74	53.0 ^b^	10.3	52.6	12.6	
Self-reported health						
Excellent/Very good	845	58.5 ^a^	11.0	58.3	15.3	<0.001 *
Fair/Poor	553	53.4 ^b^	10.7	53.5	13.7	
Intentional weight loss						
Not really/not trying to lose weight	1056	57.0 ^a^	11.2	56.7	15.3	<0.001 *
Yes/kind of trying to lose weight	345	54.3 ^b^	11.4	54.0	14.7	
Intentional weight gain						
Not really/not trying to gain weight	1179	56.2	11.4	56.2	15.1	0.303
Yes/kind of trying to gain weight	211	57.1	10.4	56.5	14.9	
Body Mass Index category						
Underweight	36	56.0 ^a,b^	10.8	57.1	15.9	0.001 *^,E^
Healthy weight	872	57.3 ^a^	11.1	56.8	15.6	
Overweight	209	54.8 ^b^	12.3	55.7	16.2	
Obese	88	53.3 ^b^	10.4	52.9	11.8	
Sleep: Getting enough sleep regularly						
Yes	731	56.8	11.0	56.5	15.7	0.140
No	629	55.9	11.3	55.4	14.2	
Sleep: Hours previous night						
8–10 h	723	57.8 ^a^	11.1	57.9	15.5	<0.001 *
6–7 h	548	55.4 ^b^	11.1	54.9	14.4	
<6 h	150	53.0 ^b^	11.3	52.1	14.3	
Sleep: Usual duration per night						
8–10 h	765	57.4 ^a^	11.2	57.2	15.3	<0.001 *
6–7 h	553	55.9 ^b^	10.7	55.6	13.9	
<6 h	92	50.8 ^c^	12.0	49.5	14.4	
Food and nutrition course in school						
Yes	848	56.6	11.1	56.4	15.1	0.575
No	514	56.3	11.1	56.4	14.8	
Importance of learning about food and nutrition in school						
Strongly agree/Agree	1110	56.9 ^a^	11.2	56.7	15.2	0.001 *
Neutral/Disagree/Strongly disagree	236	54.3 ^b^	10.6	54.6	14.4	

* *p* < 0.05: Independent samples t-test and one-way ANOVA with Tukey’s HSD post hoc test for two and three comparison groups, respectively. ^a,b,c^ Values in a column with unlike letters are significantly different. ^E^ Use with caution (due to small numbers in ‘underweight’ category).

## Data Availability

The datasets generated during and/or analysed during the current study are not publicly available due to the nature of the research and study participants not agreeing for their data to be shared with the public. Queries about data and materials should be directed to joyce.slater@umanitoba.ca.
